# Isoquinoline Alkaloids from *Coptis chinensis* Franch: Focus on Coptisine as a Potential Therapeutic Candidate against Gastric Cancer Cells

**DOI:** 10.3390/ijms231810330

**Published:** 2022-09-07

**Authors:** Sylwia Nakonieczna, Aneta Grabarska, Kinga Gawel, Paula Wróblewska-Łuczka, Arkadiusz Czerwonka, Andrzej Stepulak, Wirginia Kukula-Koch

**Affiliations:** 1 Department of Pharmacognosy with Medicinal Plants Garden, Medical University of Lublin, 1 Chodzki Str., 20-093 Lublin, Poland; 2 Department of Biochemistry and Molecular Biology, Medical University of Lublin, 1 Chodzki Str., 20-093 Lublin, Poland; 3 Department of Experimental and Clinical Pharmacology, Medical University of Lublin, 8b Jaczewskiego Str., 20-090 Lublin, Poland; 4 Department of Pathophysiology, Medical University of Lublin, 8b Jaczewskiego Str., 20-090 Lublin, Poland

**Keywords:** *Coptis chinensis* Franch, isoquinoline alkaloids, berberine, palmatine, jatrorrhizine, coptisine, CPC chromatography technique, gastric cancer, zebrafish

## Abstract

Gastric cancer (GC) has high incidence rates and constitutes a common cause of cancer mortality. Despite advances in treatment, GC remains a challenge in cancer therapy which is why novel treatment strategies are needed. The interest in natural compounds has increased significantly in recent years because of their numerous biological activities, including anti-cancer action. The isolation of the bioactive compounds from *Coptis chinensis* Franch was carried out with the Centrifugal Partition Chromatography (CPC) technique, using a biphasic solvent system composed of chloroform (CHCl_3_)—methanol (MeOH)—water (H_2_O) (4:3:3, *v*/*v*) with an addition of hydrochloric acid and trietylamine. The identity of the isolated alkaloids was confirmed using a high resolution HPLC-MS chromatograph. The phytochemical constituents of *Coptis chinensis* such as berberine, jatrorrhizine, palmatine and coptisine significantly inhibited the viability and growth of gastric cancer cell lines ACC-201 and NCI-N87 in a dose-dependent manner, with coptisine showing the highest efficacy as revealed using MTT and BrdU assays, respectively. Flow cytometry analysis confirmed the coptisine-induced population of gastric cancer cells in sub-G1 phase and apoptosis. The combination of coptisine with cisplatin at the fixed-ratio of 1:1 exerted synergistic and additive interactions in ACC-201 and NCI-N87, respectively, as determined by means of isobolographic analysis. In in vivo assay, coptisine was safe for developing zebrafish at the dose equivalent to the highest dose active in vitro, but higher doses (greater than 10 times) caused morphological abnormalities in larvae. Our findings provide a theoretical foundation to further studies on more detailed mechanisms of the bioactive compounds from *Coptis chinensis* Franch anti-cancer action that inhibit GC cell survival in in vitro settings.

## 1. Introduction

Gastric cancer (GC) is the most common cancer type of the gastrointestinal tract [1]. According to the World Health Organization, the incidence and mortality rates for this type of cancer rank fifth and fourth, respectively [2]. GC is a phenotypically heterogeneous multifactorial disease [3]. Histologically, adenocarcinoma of the stomach accounts for about 90–95% of all gastric tumors [4]. The cause of GC, like most other cancers, is not fully understood. It is assumed that environmental conditions, including diet and the *Helicobacter pylori* infection significantly increase the risk of developing GC [5,6]. Generally, GC has a poor prognosis due to its delayed diagnosis. In Europe, an early-stage GC is less than 10% of cases and is most often found by chance [7]. Surgery and perioperative chemotherapy or postoperative adjuvant chemoradiotherapy now represent the standard of care for patients with resectable GC [8]. However, the use of these conventional treatments using chemotherapy is associated with the occurrence of many side effects. Novel therapeutic regimens are currently being developed and evaluated, including targeted therapies, but they also fail because of the highly complex gene expression profile of GC [9].

With that in mind, the search for new approaches that could be used in the treatment and/or prevention of cancer is urgently needed. In recent years, there has been an increasing interest in research on phytochemicals that are able to inhibit, delay or reverse carcinogenesis [10,11,12,13]. It is worth noting that these studies have led to the identification of a number of plant- and microorganism-derived anticancer drugs that have been approved by the Food and Drug Administration and are widespread in clinical practice [14]. Moreover, there are numerous natural compounds with potential anticancer activity tested under ongoing preclinical and clinical trials [15,16], and it is not surprising that the number of newly discovered bioactive substances continues to rise.

Among plant-derived compounds exerting diverse beneficial properties, isoquinoline alkaloids should be listed. Generally, alkaloids are a class of organic nitrogen-containing small molecules which, due to their physiological properties, have become new classes of drugs. Alkaloids include neuroactive molecules (caffeine and nicotine), molecules possessing local anaesthetic properties (morphine and cocaine), blood vessel constrictors (ergonovine and ephedrine) as well as the anticancer agents such as vincristine, vinblastine, paclitaxel and camptothecin [17].

*Coptis chinensis* Franch. (Weilian in Chinese) is a small plant in the family Ranunculaceae [18] with intensely yellow, branched rhizomes, feathery leaves and small, five-petaled flowers [19]. Based on the Chinese Pharmacopoeia Edition 2020 [20], this species is one of the ingredients of a common botanical drug called *Coptidis rhizoma* (*C. rhizoma*, Chinese goldthread; Huanglian in Chinese). Accumulating studies reported a broad spectrum of pharmacologic benefits of *Coptidis rhizoma* such as antiviral, antibacterial, antifungal, effective in hepatic steatosis, antiatherosclerotic, antiarrhythmic, antihypertensive, cardioprotective, antidiabetic, anti-inflammatory, antioxidative, neuroprotective, and anticancer [21]. Various biological and pharmacological activities of extracts from *Coptis chinensis* result from the presence of alkaloids—the derivatives of phenylalanine that contain an isoquinoline moiety in their structure, namely coptisine (**11**), berberine (**13**), jatrorrhizine (**10**), magnoflorine and other protoberberines and aporphines [21]. Undoubtedly, the number of components identified in the extracts of *Coptis chinensis* is large. However, due to the marked structural similarity of all constituents, their purification from plant material for bioactivity studies is difficult and challenging.

To obtain sufficient quantities of high purity isoquinoline alkaloids such as **13**, **11**, **10** and palmatine (**12**) from the rhizomes of *Coptis chinensis*, a modern purification technique, namely Centrifugal Partition Chromatography (CPC) was used. This method is based on the separation of the molecules without solid support, but between two immiscible liquid phases—a stationary one filling the rotating column, and a mobile one—that is pumped through the column [22,23]. This feature is crucial for the isolation of compounds of natural origin, as it excludes the processes of components’ adsorption on the stationary phases. This is problematic especially in the case of alkaloids, and overcomes the problems with poor recovery rate and performance. CPC analyses are also performed using analytical grade reagents, which reduce the separation costs significantly and favor the rules of green chemistry [24].

Furthermore, our studies aimed at isolated alkaloids’ ability to inhibit the growth of GC cells in in vitro studies. For this purpose, the MTT (3-[4,5-dimethylthiazol-2-yl]-2,5 diphenyltetrazolium bromide) and bromodeoxyuridine (BrdU) incorporation assays were carried out using ACC-201 and NCI-N87 cancer cell lines as models. Studies were also conducted to assess the effects of the most active compound i.e., **11** on the cell cycle progression and induction of apoptosis. Frequently, the drug combination significantly enhances their anticancer action. Therefore, the isobolographic analysis has been applied to show the eventual synergistic or antagonistic actions of the selected alkaloid when combined with cisplatin (CDDP). In addition, we assessed the safety of **11** on developing zebrafish.

## 2. Results

### 2.1. Compositional Studies and Isolation of Alkaloids from Coptis chinensis Root Methanolic Extract by CPC Chromatography

The applied chromatographic conditions provided effective separation of metabolites from the methanolic extract from the roots of *Coptis chinensis* (See Figure S1 in the Supplementary File). High resolution mass measurements, the analysis of retention times and fragmentation patterns provided sufficient data to identify several alkaloids in the tested extract, based on a direct comparison with the scientific literature. The list of major metabolites identified in the extract is listed below in Table 1, and their MS/MS spectra are pasted to the Table S1 in the Supplementary File.

The rhizome of *Coptis chinensis* is rich in protoberberine alkaloids. They are characterized by a similar molecular structure as they originate from isoquinoline moiety. The major differences between the compounds from this class are the substituents that are attached to the four-ring system. That is why many of them are characterized by a similar fragmentation pattern. Berberine (**13**) and epiberberine (**9**) are the major components of *Coptis chinensis* rhizomes. The former compound was identified in different extracts from the representatives of Ranunculales, and can be treated as a chemotaxonomic marker of this botanical order [31]. The quantitative analysis of the total extract was performed based on the analysis of the peak area recorded by the UV detector in 290 nm. The calculations revealed that the major component of the extract among the tested isoquinoline alkaloids was berberine (**13**) with the content of 6.21 ± 0.23%, followed by palmatine (**12**) (1.62 ± 0.031%), epiberberine (**9**) (1.53 ±0.018), jatrorrhizine (**10**) (0.621 ±0.019%) and coptisine (**11**) (0.495 ± 0.0052%). The quantity calculated for berberine (**13**) and palmatine (**12**) resembled the one reported by Peng and collaborators [32], who determined their quantity as 6.88 and 1.46%, respectively. The concentration of the remaining compounds was lower in the herein tested sample, as the other authors reported 1.02% of **10** and 1.37% of **11** in their sample. The differences in the composition may result from another extraction technique or the origin of the plant.

**Figure 1:**
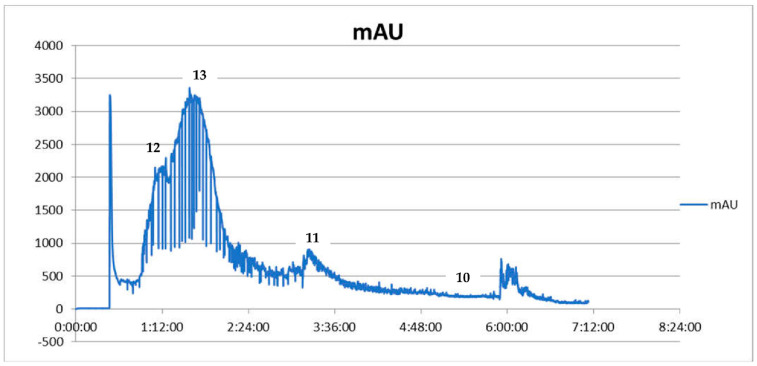
CPC chromatogram showing the fractionation of *Coptis chinensis* methanolic extract from the roots expressed as intensity units (mAU) to time (UV detection at 290 nm) (**12**—palmatine, **13**—berberine, **11**—coptisine, **10**—jatrorrhizine).

**Figure 2:**
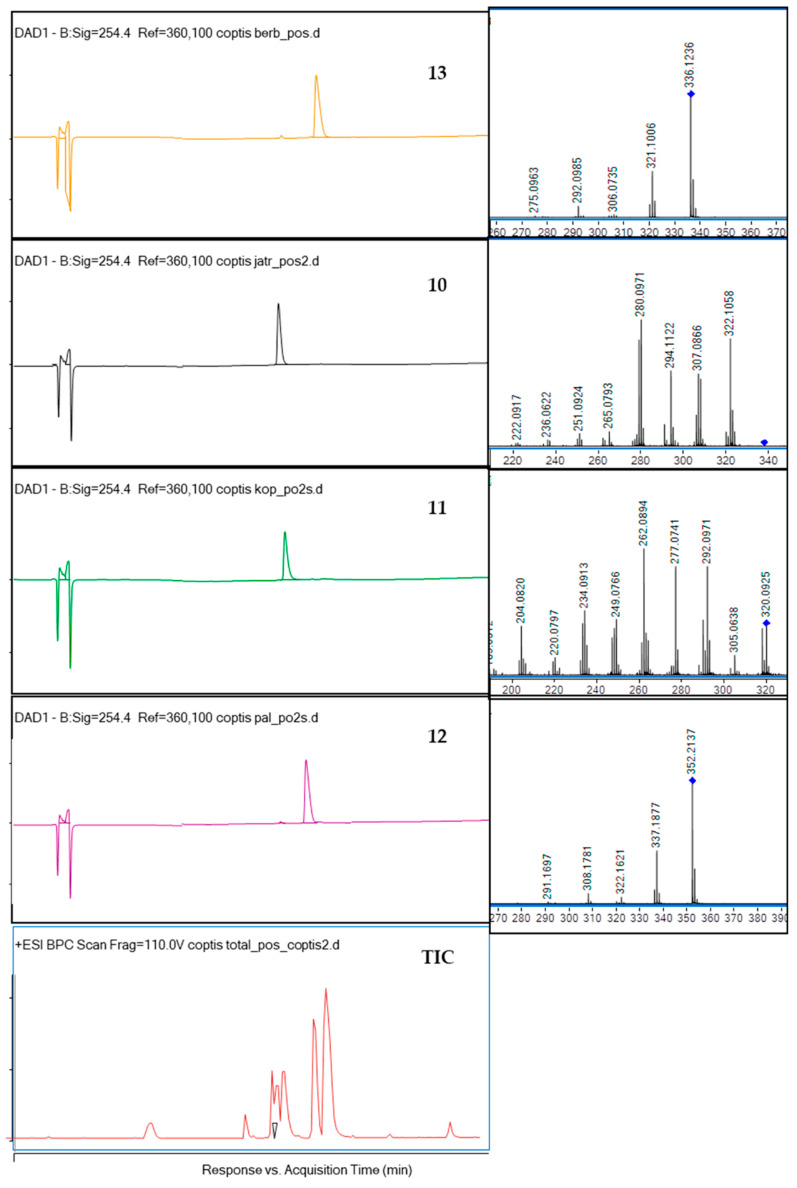
The HPLC-DAD chromatograms of the isolated compounds **13**, **10**, **11**, **12** and of the total ion chromatogram (TIC) at 254 nm together with their fragmentation spectra (on the right side) and the crude extract chromatogram.

The CPC-based fractionation of the methanolic extract from the rhizomes of *Coptis chinensis* using a chloroform-based biphasic solvent system turned out to be an efficient and repetitive technique for obtaining high purity compounds. This procedure was introduced to the study, as some reference compounds (like **11** and **10**) are expensive. Their isolation from a plant material was cheaper than the purchase and enabled the bioactivity studies. The composition of the biphasic solvent system was initially published by Sun et al. [33] and was primarily intended for HSCCC instruments that use a different operation manner—a hydrodynamic one. Following the example of his work, we applied the biphasic solvent system composed of chloroform (CHCl_3_) –methanol (MeOH)—water (H_2_O) (4:3:3, *v*/*v*) adding hydrochloric acid (HCl) to the aqueous phase and trietylamine (TEA) to the organic phase on a CPC chromatograph, optimizing the column rotation speed and solvent flow rate to a differently constructed column. As a consequence of the performed optimization, four alkaloids with high purity were isolated from the crude extract by CPC chromatograph and were identified by HPLC-ESI-QTOF-MS/MS as **13**, **10**, **11** and **12**. The separation efficacy of the performed analysis was similar to the one published by Sun et al. [33], however, the compounds of interest were eluted much faster—after 300 min—than by the other authors, who performed the separation for 11 h. The difference was due to the application of a faster flow rate (5 mL/min in comparison with 2 mL/min of other studies) and faster rotation speed of the column (1050 rpm instead of 850 rpm). The dominant component in *Coptis chinensis* methanol extract was **13**. Other alkaloids (**10**, **11**, **12**) made up smaller proportions of the methanol extract of *Coptis chinensis*. The first separation was performed using a 500 mg portion of the extract (see Figure 1). As the used biphasic solvent was found to be selective for coptisine (**11**), in order to obtain higher quantities of **11** for bioactivity studies, the following runs were performed with more than 2 g of extract dissolved in 10 mL of the system. As a result, 9–12 mg of **11** were obtained in one purification protocol, which was found to be financially beneficial. Unfortunately, columbamine (8) was not obtained in the applied conditions. It is worth noting that the applied conditions led to a successful recovery of high purity alkaloids from the extract. The purity of the final isolated compounds is presented in Figure 2, exceeding 95%, and the chromatogram recorded during the separation procedure is presented in Figure 1.

Previously, the fractionation of *Coptis chinensis* extracts by counter-current instruments was also described by other authors. Yang and collaborators [34] operated a high-speed counter-current chromatograph in a solvent system composed of chloroform: methanol: water at the ratios between 4:3:2 (*v*/*v*/*v*) and 4:1.5:2 (*v*/*v*/*v*), respectively, with an addition of hydrochloric acid to the system. Yang et al. [34] underlined that together with a decrease in methanol (from 3 to 1.5 parts) and hydrochloric acid contents (from 0.3 to 0.1 M), a prolongation of the alkaloids’ retention times was observed. Several trials to introduce their system into a CPC chromatograph were found to be not successful enough. That is why the authors decided on the addition of TEA to the upper phase (as described by Sun and co-investigators [25]) and to conduct the separation in the pH-zone refining mode. A similar solvent system selectiveness in the recovery of isoquinoline alkaloids from *Coptis chinensis* to the herein proposed method was described by Peng and collaborators [32]. These researchers also obtained berberine (**13**), palmatine (**12**), jatrorrhizine (**10**) and coptisine (**11**) on an HSCCC chromatograph within 240 min, applying the rotation speed of 850 rpm and the flow rate of 2 mL/min and using a biphasic solvent system composed of n-hexane: ethyl acetate: methanol: 1% acetic acid (1:1:1:1 *v/v/v/v*). As described by Zhang and collaborators [35], a typical quaternary solvent system from the group of HEMW at systems was found efficient in the separation of isoquinolines from *Coptis chinensis* rhizomes. The application of a mixture of n-hexane: ethyl acetate: methanol: water (2:5:2:5 *v/v/v/v*) on an HSCCC chromatograph resulted in the isolation of berberine (**13**), epiberberine (**9**), jatrorrhizine (**10**), worenine (**18**) and coptisine (**11**) within 5 h. The flow rate of the upper mobile phase and the rotation speed of the column within the first 2.5 h were set at 2 mL/min and 850 rpm, whereas for the remaining 2.5 h at 5 L/min and 650 rpm, respectively. The separation efficiency of coptisine (**11**) in this protocol was slightly lower (the final purity of 88.5%), however epiberberine (**9**) and worenine (**18**) were obtained as high purity compounds (purity exceeding 95%). The protocols described above show some potential for future application on CPC chromatographs. For the moment the fractionation of *Coptis chinensis* extract on CPC chromatographs was only performed by Kim [36] who used a mixture of n-butanol: acetic acid: water (4:1:5 *v/v/v*) to obtain high purity berberine (**13**) from 80% methanolic extract in the ascending mode. The injection of 324 mg of buthanol fraction from the extract on a CPC chromatograph and then on a preparative chromatograph provided 16.8 mg of high purity alkaloid.

Further optimization studies should be performed on hydrostatic instrumentation to introduce the other protocols into laboratory practice, as they could help isolate other constituents of *Coptis chinensis* extract, like worenine (**18**), columbamine (**8**) or epiberberine (**9**).

The identification of four isolated alkaloids was performed based on the comparison of their retention times with reference compounds, and based on the analysis of MS/MS spectra. The fragmentation pattern (MS/MS spectrum) of berberine (**13**) presented in Table S1 in the Supplementary File and in Figure 2 shows the detachment of several moieties linked to the isoquinoline system like methoxyl, methylene, hydroxyl and methyl groups, similar to other isoquinoline alkaloids. In the spectrum fragments with the *m/z* value of 28 Da (for CO group), 18 Da (for the detachment of H_2_O), or 15 Da (for CH_3_) can be observed. Berberine (**13**) molecule contains two-methoxyl groups substituted at C9 and C10 whose detachment is represented by *m/z* 320.0928, and *m/z* 306.0735 signals, respectively. The ion at *m/z* 321.1006 was determined as the neutral loss of the radical group CH_3_ from C9 or C10, and the ion at *m/z* 320.0928 was produced by the loss of one CH_4_ group. Then, the carbonyl group can be formed in the C9 or C10 position of D ring via the molecule rearrangement. Also, the ion at *m/z* 292.0985 was produced by the loss of CH_4_ and CO.

The fragmentation of coptisine (**11**) resulted in the formation of the *m/z* signal at 292.0971 from the detachment of CO, 277.0741—from the additional loss of methyl group (CH_3_), 262.0894—from the loss of another CH_3_ moiety, 249.0766—from the detachment of CO group from *m*/*z* of 277.0741, and 234.0913—from the loss of another CO group from 262.0894 [37]. Previous reports on the fragmentation of protoberberine alkaloids in the ionization source suggest that one of the molecular transformations that occurs in these types of alkaloids as berberine (**13**) is the subsequent loss of a methyl radical and CO. The same behavior was observed in the case of palmatine (**12**) and jatrorrhizine (**10**)—the other protoberberine alkaloids—with methoxy groups attached at C4, C5 or C9, C10 carbon atoms. According to Sun and co-investigators [37], the CH_4_ group—if present in a molecule—is the first one to be detached in the ESI ionization type, and loss of the CO group is observed in the molecules like coptisine (**11**), where methoxyl substituents are not present. Following this pathway, jatrorrhizine (**10**) was identified based on the 322.1058 *m/z* signal from the loss of the methyl group, 294.1122, 308.0085, 306.0272 and 280.0171 *m/z* signals from the losses of three methyl groups, two methyl groups, two methyl groups and two protons, and two methyl groups and CO, according to the previously published results [38].

Spectra of palmatine (**12**) confirmed a similar fragmentation pattern as of other isoquinolines [39], namely the detachment of CH_3_ (signal at 337 *m/z*), CH_2_O (322 *m/z*), CO from the 336 *m/z* (308), and CO from 322 (294).

### 2.2. Impact of Isoquinoline Alkaloids Isolated from Coptis chinensis on the Viability and Proliferation of ACC-201 and NCI-N87 Gastric Cancer Cells

**Figure 3:**
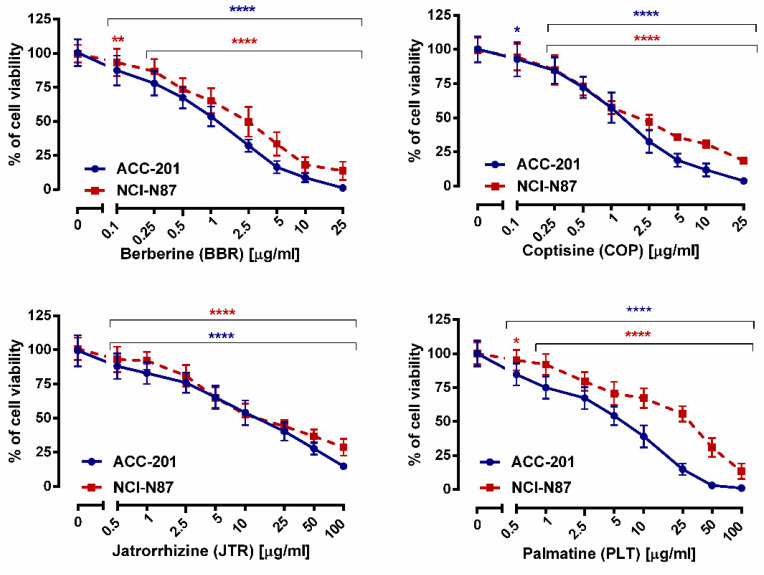
The effect of isoquinoline alkaloids isolated from *Coptis chinensis* on the viability of human GC cell lines was measured by MTT assay after 72 h. Results are presented as mean ± SD at each concentration. (* *p* < 0.05; ** *p* < 0.01; **** *p* < 0.0001 vs. control group; Tukey’s *post-hoc* test), n = 24 per concentration from three independent experiments.

The first step in the current study was to determine the effect of berberine (**13**), coptisine (**11**), jatrorrhizine (**10**) and palmatine (**12**), the major compounds of *Coptis chinensis*, on the viability of ACC-201 and NCI-N87 GC cells. All studied alkaloids decreased this parameter in a concentration-dependent manner, as revealed in MTT assay (Figure 3).

We found that **10** exhibited the weakest cytotoxicity towards GC cells with IC_50_ values varying from 12.15 µg/mL (35.90 µM) to 17.85 µg/mL (52.75 µM), depending on the cell line. On the other hand, **13** and **11** showed similar characteristics of viability inhibition of both ACC-201 and NCI-N87 cancer cells. The IC_50_ values of **13** and **11** were 0.999 µg/mL (2.97 µM) and 1.260 µg/mL (3.93 µM) for ACC-201 cells, respectively. Meanwhile, the mentioned alkaloids showed IC_50_ in the range of 2.023–2.110 µg/mL (6.01–6.58 µM) in NCI-N87 cells. The **12** exerted the higher capacity to inhibit cell viability towards ACC-201 cells (Table 2). Observed differences in the effectiveness of **12** targeting GC cells could be related to genetic differences among these cell lines.

**Figure 4:**
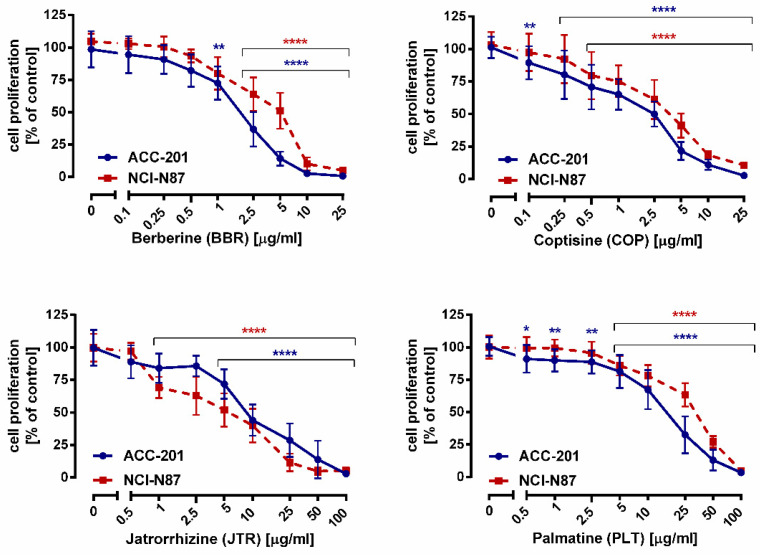
The effect of isoquinoline alkaloids isolated from *Coptis chinensis* on the proliferation of human gastric cancer cell lines was measured by BrdU assay after 72 h. Results are presented as mean ± SD at each concentration. (* *p* < 0.05; ** *p* < 0.01; **** *p* < 0.0001 vs. control group; Tukey’s *post-hoc* test), n = 24 per concentration from three independent experiments.

The effects of alkaloids on GC cells growth were also monitored using a more sensitive and specific BrdU assay (Figure 4). A significant inhibition of DNA synthesis in response to **13**, **11**, **10**, and **12** has been observed, as evidenced by a decrease in the incorporation of BrdU into DNA.

### 2.3. Coptisine Treatment Increases Population of Cells in Sub-G1 Phase and Induces Apoptosis in Gastric Cancer Cell Lines

**Figure 5:**
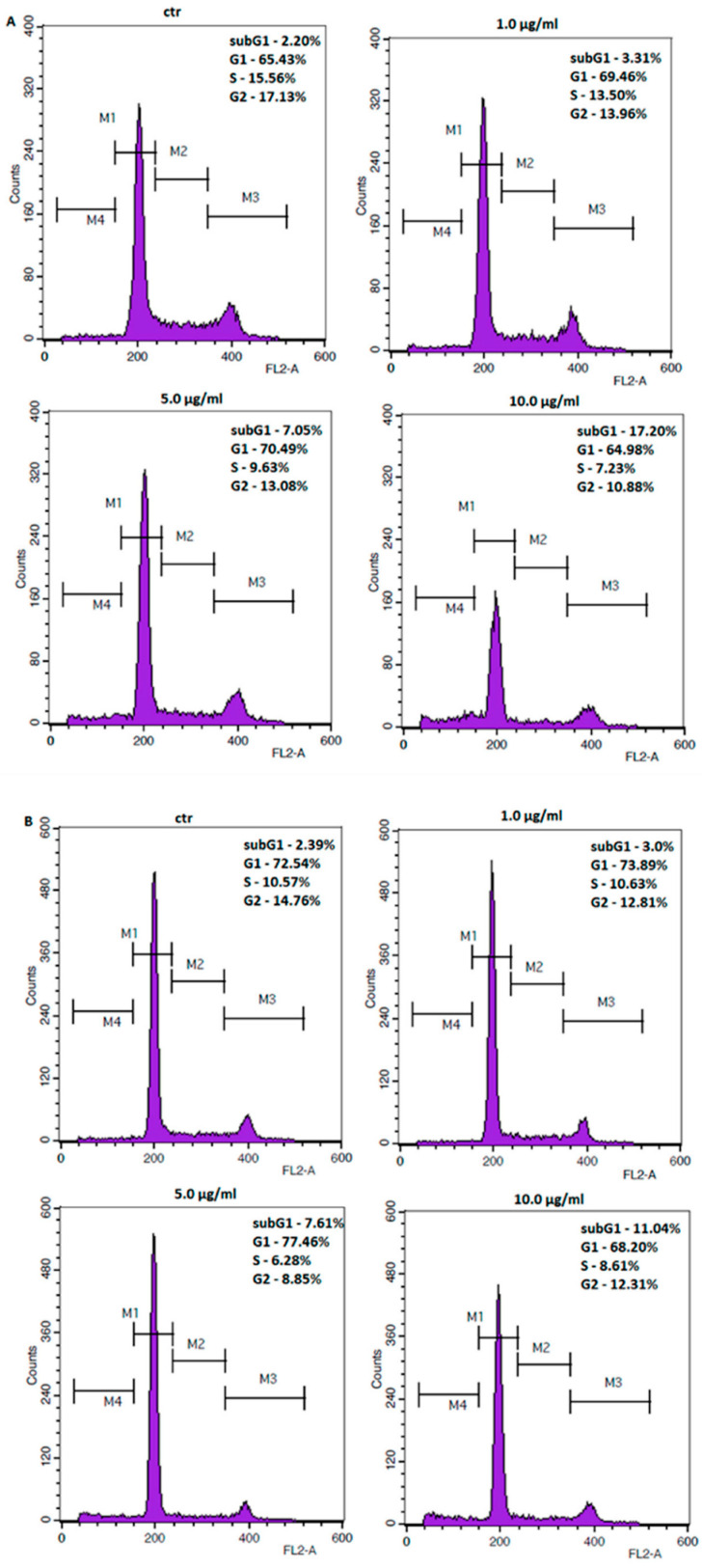
Representative flow cytometry histogram peaks of the ACC-201 (**A**) and NCI-N87 (**B**) gastric cancer cell lines after the treatment with a medium (ctr) and coptisine (**11**). Region M1, M2, M3 and M4 included G1, S, G2 and subG1 phase, respectively.

GC cell lines were treated with increasing concentrations of **11** to assess its potential impact against the cell cycle changes and cell death. Flow cytometry analysis showed that the number of cells with sub-G1 DNA content increased significantly from 2.20% at control to 17.20% at 10 μg/mL (31.21 µM) of **11,** and from 2.39% at control to 11.04% at 10 μg/mL (31.21 µM) of **11** in ACC-201 and NCI-N87 cells, respectively (Figure 5). Accumulation of cells in the subG1 phase may suggest induction of apoptosis.

**Figure 6:**
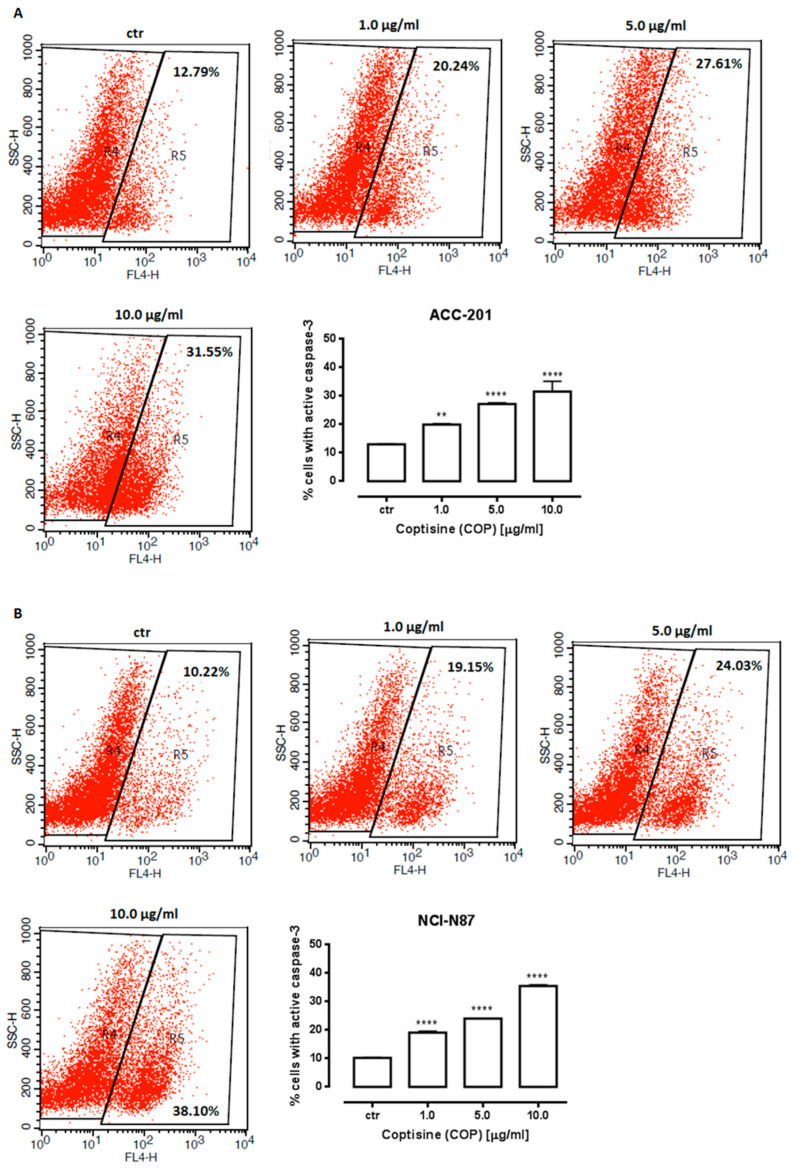
Representative flow cytometry dot plot graphs of ACC-201 (**A**) and NCI-N87 (**B**) gastric cell lines after the treatment with a medium (ctr) and coptisine (**11**). Region R5 included apoptotic cells with active caspase-3. Results are presented as mean ± SD at each concentration (** *p* < 0.01; **** *p* < 0.0001 vs. control group; Tukey’s *post-hoc* test), n = 5 per concentration from three independent experiments.

Therefore, an additional method was used to confirm apoptotic cell death. Cellular apoptosis was analyzed by the measurement of caspase-3/7, which acts as an effector or “executioner” caspases leading to the final stages of programmed cell death [40]. The results showed that the number of cells with activated caspase-3/7 significantly increased in response to **11** treatment (Figure 6). When exposed to 10 µg/mL (31.21 µM) of **11**, the percentage of apoptotic cells reached 31.55% ± 1.809 and 35.55% ± 0.1889 in ACC-201 and NCI-N87 cells, respectively.

### 2.4. The Anti-Proliferative Effects of Coptisine Administered in Combinatin with Cisplatin

**Figure 7:**
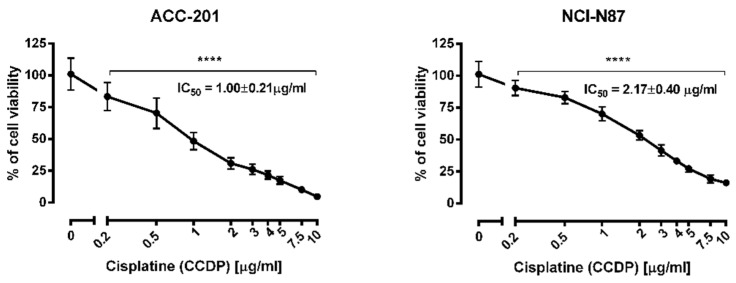
The effect of cisplatin (CDDP) on the viability of human gastric cancer cell lines was measured by MTT assay after 72 h. Results are presented as mean ± SD at each concentration. (**** *p* < 0.0001 vs. control group; Tukey’s *post-hoc* test), n = 24 per concentration from three independent experiments.

CDDP administered alone dose-dependently reduced the viability of ACC-201 and NCI-N87 cells with an IC_50_ value of 1.00 and 2.17 µg/mL, respectively (Figure 7).

**Figure 8:**
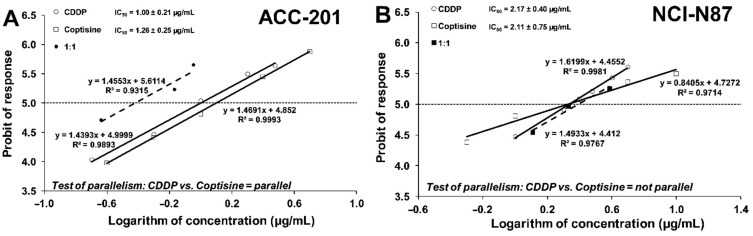
Log-probit dose–response relationship curves (DRRCs) for cisplatin (CDDP) and coptisine (**11**) administered alone, and in combinations at the fixed ratio of 1:1 (dotted line), illustrating the anti-proliferative effects of the drugs in the human gastric cancer cell lines ACC-201 (**A**) and NCI-N87 (**B**) measured in vitro by the MTT assay.

Test for parallelism of two dose–response relationship curves (DRRCs) between CDDP and **11** performed according to Litchfield and Wilcoxon [41] revealed that the DRRCs of both compounds were parallel and non-parallel to each other in ACC-201 and NCI-N87 cancer cell lines, respectively (Figure 8).

### 2.5. Isobolographic Analysis of the Interactions between Cisplatin and Coptisine

**Figure 9:**
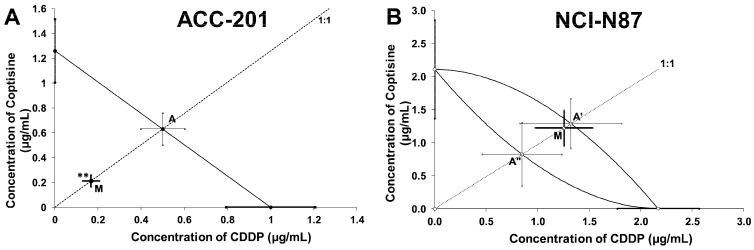
Type I isobolographic analysis for parallel (**A**) and non-parallel (**B**) dose–response relationship curves (DRRCs) between cisplatin (CDDP) and coptisine (**11**) at the fixed-ratio of 1:1 in gastric cancer cell lines measured in vitro by the MTT assay. ** *p* < 0.01 vs. IC_50add_.

Type I isobolographic analysis was performed for parallel and non-parallel DRRCs. The point A (Figure 9A) and the points A’ and A” (Figure 9B) depict the theoretically calculated IC_50add_ values. The point M (Figure 9A,B) represents the experimentally-derived IC_50 exp_ value for the total dose of the mixture, expressed as proportions of CDDP and **11** that produced a 50% anti-proliferative effect in the ACC-201 and NCI-N87 cancer cell lines measured in vitro by the MTT assay.

On the graph 9A, the point M value is placed significantly (Welch’s *t* test: *t* = 3.037; df = 295.3; *p* = 0.0026) below the point A, indicating a synergistic interaction between CDDP and **11** in ACC-201 cell line (Figure 9A, Table 3). The analysis of interaction for the non-parallel concentration-response effects in the NCI-N87 cell line, in turn revealed that the combination of CDDP and **11** exerted an additive interaction. The experimentally-derived IC_50_ mix value is placed close to the point A’ (Figure 9B, Table 4).

### 2.6. Effect of Coptisine on Developing Zebrafish

**Figure 10:**
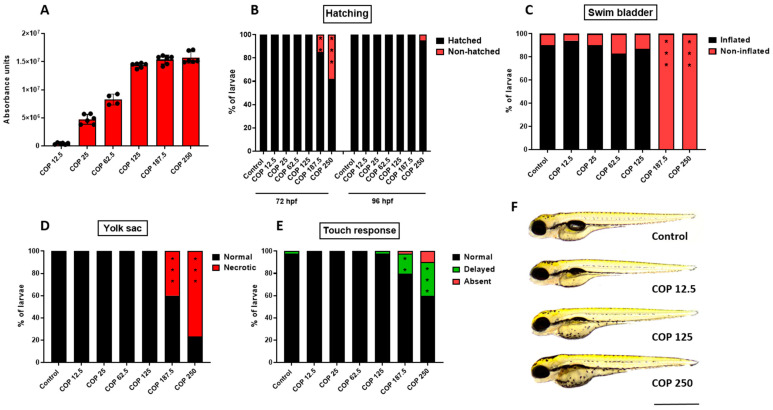
(**A**) The absorbance of different doses of coptisine (**11**) (12.5, 25, 62.5, 125, 187.5 or 250 µg/mL) (39.02, 78.04, 195.12, 390.24, 585.37 or 780.49 µM) by larval zebrafish after 95 h of incubation, n = 4–6 samples/group, n = 100 larvae per sample. Dose-dependent effect of coptisine (**11**) on (**B**) hatching rate (%) of 72 and 96 hpf-old larvae, n = 20–24/group; (**C**) swim bladder inflation at 96 hpf, n = 30–34/group; (**D**) yolk sac necrosis at 96 hpf, n = 30–34/group; (**E**) touch-evoked response at 96 hpf, n = 30–34/group; (**F**) representative images of larvae exposed to coptisine (**11**) (12.5, 125 or 250 µg/mL) (39.02, 390.24 or 780.49 µM). All experiments were done in triplicates and data were pooled together (** *p* < 0.01; *** *p* < 0.001 vs. control group, Fisher’s exact test). COP-coptisine (**11**), hpf—hours post-fertilization. Scale bar 1 mm.

In order to evaluate the safety of **11** in vivo, the zebrafish acute toxicity assay was carried out according to the Organization for Economic Cooperation and Development (OECD) guideline for the testing of chemicals (Test NO. 236) [42]. Therefore, zebrafish embryos starting at 1 h post-fertilization (hpf) were incubated for 95 h, in different doses of **11** (12.5, 25, 62.5, 125, 187.5 or 250 µg/mL) (39.02, 78.04, 195.12, 390.24, 585.37 or 780.49 µM). The lowest **11** dose was equivalent to the highest in vitro dose (12.5 µg/mL) (39.02 µM), while the maximal dose used was 20 times higher (250 µg/mL) (780.49 µM). First, we showed that **11** is absorbed by developing zebrafish, with the dose of 125 µg/mL (390.24 µM) being the loading dose (Figure 10A). None of the doses used substantially affected the hatching of larvae at 96 hpf, however compared to control group, at 72 hpf doses of 187.5 (585.37 µM) and 250 µg/mL (780.49 µM) delayed hatching of 15% (*p* < 0.01) and 38% (*p* < 0.001) of larvae, respectively (Figure 10B). Phenotypic scoring revealed that up to 125 µg/mL (390.24 µM) **11** did not exert any meaningful morphological abnormalities, thus one may assume it is safe for developing zebrafish (*p* > 0.05; Figure 10C–E; for representative image see Figure 10F). However, larvae incubated with 187.5 and 250 µg/mL (585.37 and 780.49 µM) lacked a swim bladder (*p* < 0.001), and yolk sac necrosis was observed in 40 and 77% (*p* < 0.001) of larvae exposed to the above-mentioned doses. In the touch response assay, which allows the assessment of muscle performance, a delayed response was observed in 18% (*p* < 0.01) and 30% (*p* < 0.001) of fish (187.5 and 250 µg/mL, respectively) (585.37 and 780.49 µM, respectively). We did not observe any other morphological changes (i.e., jaw malformations, pericardial oedema, body curvature, yolk sac oedema, hemorrhage) in any of the doses used (for representative images see Figure 10F).

## 3. Discussion

The rhizomes of *Coptis chinensis* are rich sources of different types of isoquinoline alkaloids, that had been proved to inhibit the development of cancer cells in various in vitro and in vivo models of different types of cancer [43,44,45,46,47]. However, to perform the experimental studies, pure compounds are necessary. To recover structurally similar alkaloids from the rhizomes of Chinese goldthread, CPC was applied on the crude dried extract.

The herein used purification method was a modification of the previously published protocol [20] suitable for the HSCCC chromatographs. In this study, the methodology was adjusted for the application on CPCs. As a result, it provided high purity **13**, **12**, **10** and **11** from the methanolic extract of dried rhizomes of *Coptis chinensis* in the analysis that lasted for 400 min. The compounds of concern were obtained in the eluate from the CPC column already after 300 min, which is a marked achievement, having in mind only one rotation axis of CPC chromatographs. The applied novel purification technique favors the recovery of alkaloids from crude extracts. Nitrogen-containing compounds often adsorb on stationary phases and co-elute with one another, due to structural similarities when isolated using traditional separation techniques. CPC that uses no solid support is characterized by a high recovery rate and mild operation conditions which can improve the purification efficiency of alkaloids [48,49]. In addition, counter-current chromatography is eagerly selected to upscale the purification protocols. Because of this fact, we selected CPC instrumentation to be able to use preparative columns in the future for the isolation of sufficient quantities of alkaloids for in vivo studies.

Our studies revealed two candidates—**13** and **11** as the most promising compounds, which exhibited a higher cytotoxic effect on GC cell lines with the lowest IC_50_ values in comparison to **12** and **10**. These results are consistent with previous studies indicating anticancer activity of **11** and **13** against hepatoma and leukemia cells in vitro [50]. It was hypothesized that the benzylisoquinoline alkaloids induced an anti-proliferative effect due to the direct inhibition of the activity of topoisomerase I, mediated by the five-membered rings located on both ends of **13** and **11** [51]. Berberine (**13**) and coptisine (**11**) share the same molecular skeleton divided into four rings, A, B, C, D with a methylenedioxyl group at C2 and C3 on the A ring and small differences in substituent patterns in the D ring [52]. In the berberine (**13**) structure, C9 and C10 of the D ring are each attached to a methoxyl group, whereas the D ring of **11** forms a methylenedioxyl group [53]. From the view of the structure—activity relationship, it has been shown that a methylenedioxy group of **13** and **11** compared to dimethoxyl group of **12** at the 2,3-positions on the ring A is important among others for anticancer activity [54], and markedly improves the inhibitory effect of **13** and **11** on cancer cell proliferation [55,56]. Different cytotoxicity responses of cancer cells to **13** and **11** may result from the fact that these alkaloids, probably due to differences in the structure, affect either common signaling pathways or have their own specific mode of action like berberine (**13**). It was found that this alkaloid uniquely regulates gene expression implicated in the mitogen activated protein kinase cascade that is frequently altered in cancer cells [55,57].

Considerable evidence exists demonstrating the promising role of **11** in cancer prevention and/or therapy. In recent years, it has been shown that **11** exerted anti-proliferative activities against pancreatic cancer (MiaPaCa-2, Panc-1) cells, hepatoma (HepG2, Hep3B, SK-Hep1, and PLC/PRF/5), leukemia (K562, U937, P3H1, and Raji) and osteosarcoma (MG63) cells [21].

To the best of our knowledge, no studies related to the ability of **11** to inhibit the growth of GC cells have been published. We reported here that **11** significantly inhibited the proliferation of ACC-201 and NCI-N87 GC cells with IC_50_ values of 1.26 µg/mL (3.93 µM) and 2.11 µg/mL (6.58 µM), respectively. The IC_50_ values obtained in our studies were significantly lower than those presented in published reports for osteosarcoma (12.99–28.54 µM), pancreatic (100 µM), as well as for lung cancer (18.09–21.60 µM) [58,59,60]. In agreement with previous findings [59,61,62], triggering of apoptosis was found to be an important mechanism in the antiproliferative activity of **11** against lung, hepatocellular and colon cancer cells. The induction of apoptosis by **11** was characterized by, among others, the activation of caspase-3, the cleavage of poly adenosine diphosphate ribose polymerase, the upregulation expression of pro-apoptotic Bax protein and the downregulation of the expression of anti-apoptotic Bcl-2 protein. Our present results on GC cells showed a similar phenomenon—the activation of caspases-3/7 in the apoptotic response of ACC-201 and NCI-N87 cells to coptisine (**11**).

In vivo studies demonstrated that the **11** treatment significantly reduced tumor volume and weight using the xenograft mouse model of hepatoma and colorectal cancer. Equally, it should be noted that no evidence of coptisine (**11**)-related toxicity was observed after the oral administration with a dosage not exceeding 150 mg/kg/day [61,63,64]. The other acute toxicity assays showed that the median lethal dose (LD_50_ value) of **11** was about 880 mg/kg on mice [65,66]. In our present toxicity-related studies using the zebrafish animal model, we demonstrated that **11** was absorbed by zebrafish embryos and larvae after a 95 h long incubation, with the dose of 125 µg/mL (390.24 µM) being the loading dose. Our observation that **11** is absorbed by larval zebrafish is in agreement with Li et al. (2014) [67] who additionally showed that **11** is metabolized to 4 different metabolites in adult zebrafish. In our case, **11** in the lower dose i.e., 12.5 µg/mL (39.02 µM) corresponding to the highest in vitro dose, seemed to be safe for developing embryos and larvae without any changes in scored parameters. Interestingly, Hu et al. (2017) [68] indicated that 11 in the dose of 10 µg/mL (31.21 µM) reduced lipid peroxidation and reactive oxygen species production as well as cell death in AAPH-exposed larval zebrafish. For comparison, in our study the dose of 125 µg/mL (390.24 µM), which was 10 times higher than the corresponding maximal dose used for in vitro assays, only mildly affected larval morphology (swim bladder was not inflated in all fish). Only the application of higher concentrations of 187.5 and 250 µg/mL (585.37 and 780.49 µM) caused significant malformations (lack of swim bladder, swim bladder necrosis, delayed touch response) in larvae after a 95 h long incubation. Although we did not measure concentrations of metabolites in larvae exposed to higher doses of **11**, taking into account the data of Li et al. (2014) one cannot rule out that observed morphological changes are the result of the accumulation of **11** metabolites but not the parent compound. Thereby, the doses used in our experiments should not develop toxicity-related changes in normal cells, as demonstrated on developing embryo and zebrafish larvae.

The other drug used in our study, cisplatin (CDDP), belongs to the class of platinum-containing chemotherapeutic agents widely applied in the treatment of solid tumors, including advanced GC [69]. The mechanism of action of CDDP has been associated with the ability to bind to DNA to form DNA adducts, thus interfering with DNA repair and leading to ultimate cell death. Many studies reported that CDDP induces toxicity such as nausea, nephrotoxicity, cardiotoxicity, hepatotoxicity and neurotoxicity [70]. In addition, cancer relapses of patients who initially responded to treatment due to drug resistance seem to be the most clinically challenging. These limitations incline studies toward the development of new therapies based on natural products combined with conventional clinically-used chemotherapeutic drugs. The clinical goal of the combination treatment is to reduce the dose of the active drug while preserving its efficacy or reducing toxicity [71]. Numerous studies both on in vitro and in vivo models of cancer have reported potentiation of the activity of clinical drugs such as CDDP, 5-fluorouracil, doxorubicin, paclitaxel, gemcitabine, and imatinib by natural compounds e.g., alkaloids, terpenoids, steroids, polyphenols, and flavonoids, including our previous reports [48,72,73,74]. These findings suggest that a combination of drugs may have synergistic or additive effects. It is well established that the beneficial effect of the interaction of dietary phytochemicals with anticancer agents might be generated among others by sensitization of cancer cells, reversing chemoresistance, and the promotion of repair mechanisms [75].

It has been observed that co-treatment of **13** and CDDP improved the sensitivity of breast cancer cells to CDDP through up-regulation of caspases 3 and 9, down-regulation of the expression of the Bcl-2 protein, increasing CDDP-induced DNA damage while reducing the level of cellular Proliferating Cell Nuclear Antigen [76]. To date, there is no record of a combinatorial regimen of **11** with CDDP against cancer. However, taking into account the similar cytotoxicity of **11** in relation to **13**, one would also expect satisfactory results of its action against GC cells in combination with CDDP. Indeed, by means of isobolographic analysis, our results showed that the combination of **11** with CDDP at the fixed-ratio of 1:1 exerted synergistic and additive interactions in ACC-201 and NCI-N87 GC cell lines, respectively. The possibility of reducing the toxicity of an already approved chemotherapeutic agent by reducing the dose and maintaining similar therapeutic properties, raises hopes for the clinical use of **11** together with CDDP in the future.

## 4. Materials and Methods

### 4.1. Reagents

The reagents, such as methanol, chloroform, hydrochloric acid and triethylamine, used for the extraction and chromatographic separation of alkaloids, were of analytical grade and were purchased from Avantor Performance Materials (Gliwice, Poland). Spectroscopic grade solvents used for the HPLC-MS analyses—acetonitrile, water and formic acid were manufactured by Merck (Darmstadt, Germany). All cell culture reagents, standards of berberine (**13**), coptisine (**11**), jatrorrhizine (**10**) and palmatine (**12**) (purity exceeding 95%) were obtained from Sigma-Aldrich (St. Louis, MO, USA).

### 4.2. Plant Material

Dried and powdered rhizomes of *Coptis chinensis* were purchased from “Nanga” herbal wholesaler (Złotów, Poland) in September 2020.

### 4.3. Extraction

Ten grams of the dried and powdered plant material were extracted with 100 mL of methanol. The extraction was performed three times, 30 min each, at room temperature using an ultrasonic bath. Next, the extracts were centrifuged for 10 min at 3500 rpm and the collected supernatants were joined and evaporated to dryness at 45 °C, using an Eppendorf Concentrator Plus evaporator (Hamburg, Germany). The obtained samples were refrigerated at 4 °C until further investigations.

### 4.4. Qualitative and Quantitative HPLC-MS Analyses

The compositional analysis of the extracts was performed using an analytical platform HPLC-ESI-QTOF-MS/MS in an optimized chromatographic method. An Agilent Technologies (Santa Clara, CA, USA) instrument composed of HPLC chromatograph with a mass detector was used in the studies. The chromatograph (1200 Series) was composed of a binary pump, a degasser, an autosampler, a thermostate, a PDA detector and a mass spectrometer—QTOF with ESI ionization (G6530B).

The separation was performed using an RP-18 Zorbax Eclipse Plus chromatographic column with dimensions of 150 mm × 2.1 mm and particle size of 3.5 µm (Agilent Technologies, Santa Clara, CA, USA) in the following gradient of acetonitrile with 0.1% formic acid (A) in 0.1% formic acid: 0 min—10% A, 17 min—32%, 18–19 min—95%, 20 min—10%. The length of the run was set at 30 min, the post time at 2 min, the injection volume at 2 µL, the flow rate at 0.200 µL/min, the temperature of the thermostat at 25 °C and the UV detection at 254, 290 ad 365 nm. The following settings of the mass detector were applied: the *m*/*z* range of 50–1000 u, the MS scan rate of 1 spectrum/s, collision energies of 20 and 40 V, gas temperature of 300 °C, sheath gas temperature of 325 °C, gas flows of 12 L/min, capillary voltage of 3000 V, fragmentor voltage of 110 V, skimmer voltage of 65 V, nebulizer pressure of 35 psig.

The proposed identification of metabolites present in the positive ionization mode was based on the high-resolution *m/z* measurement, retention time, fragmentation pattern, data from scientific literature and open spectral databases (Metlin).

The quantitative analysis was performed on the total methanolic rhizome extract to provide information about its composition. Firstly, the calibration curves of standards of isoquinoline alkaloids (**13**, **11**, **10** and **12**) were purchased from Sigma-Aldrich (St. Louis, MO, USA) and the stock solutions of every compound at a concentration of 1 mg/mL were prepared. Later, 5 different dilutions of every standard were obtained from the stock solution to form a range of concentrations between 0.005–1 mg/mL. Every solution was injected at the volume of 2 µL in the same method as for the analysis of the extract. Quantitative data were collected from respective calibration curve equations drawn for every standard compound—from a triple injection of the total extract. The following calibration equation curves were obtained with regression values (R^2^): for **13** y = 6475x + 206.3 (R^2^ = 0.9901), for **12** y = 6875x − 33.7 (R^2^ = 0.9995), for **11** y = 6295x − 111.7 (R^2^ = 0.9998), for **10** y = 6384x − 133.8 (R^2^ = 0.9965).

### 4.5. Fractionation of Extract by Centrifugal Partition Chromatography

The fractionation protocol by Sun and collaborators [33] was adjusted to the use in the CPC chromatograph and used in the study. First, the biphasic solvent system composed of chloroform (CHCl_3_)—methanol (MeOH)—water (H_2_O) (4:3:3, *v*/*v*) [33] was equilibrated in a separatory funnel, and the two phases were separated before use. The upper aqueous phase (the stationary phase) was acidified with HCl at the concentration of 60 mM, and the lower organic phase (the mobile phase) was rendered basic by adding triethylamine (TEA) at the concentration of 5 mM. Next, 0.5 g of the obtained extract was dissolved in the 40:60 (*v*/*v*) mixture of the acidified upper phase and neutral lower phase. The separation was conducted on a hydrostatic CPC in the ascending mode of separation. First, the chloroform-containing lower stationary phase with an addition of TEA was introduced on the column at the speed of 20 mL/min with 1050 rpm. Then, the extract was injected together with the mobile phase and was fractionated by an alkalified upper aqueous phase at 1050 rpm and a flow rate of 5 mL/min. Twelve milliliter volume fractions were collected by the fraction collector and the analysis lasted 410 min. During the run, the UV absorption of the eluate was monitored at 254 and 290 nm.

After the run, 2 mL of each purified fraction was filtered through a nylon syringe filter (0.2 µm pore size) to autosampler vials, evaporated to dryness using an Eppendorf Concentrator Plus evaporator (Hamburg, Germany), re-dissolved in methanol and subjected to HPLC-MS analysis in the method described above. Pure isoquinoline alkaloids from the separation were used for the bioactivity studies. The separation was necessary to obtain sufficient quantities of alkaloids (especially **10** and **11**) for biological studies due to high prices of reference compounds.

### 4.6. Cell Lines Culture

The human GC cell line ACC-201 was obtained from the Leibniz Institute DSMZ-German Collection of Microorganisms and Cell Cultures. The human GC cell line CRL-5822 (NCI-N87) was obtained from the American Type Culture Collection (ATCC). Both cancer cell lines were cultured in RPMI1640 medium supplemented with 10% FBS (Sigma-Aldrich) and antibiotics (100 IU/mL of penicillin and 100 μg/mL of streptomycin (Sigma-Aldrich) in a humidified 5% CO_2_ atmosphere at 37 °C (ACC-201 and NCI-N87).

### 4.7. Cell Viability Assay

A colorimetric MTT assay was used to assess the cell metabolic activity as an indicator of cell viability. The MTT test involves the conversion of yellow dye MTT to purple formazan by the mitochondrial enzyme [77]. Briefly, isolated alkaloids were dissolved in DMSO and stored in aliquots at −20 °C prior to use. The ACC-201 and NCI-N87 cells were seeded in 96-well plates (Nunc, Rochester, NY, USA) at a density of 2 × 10^4^ cells/mL and 1 × 10^5^ cells/mL, respectively. After overnight attachment, the cells were treated with different concentrations of alkaloids for 72 h. Next, the cells were incubated with MTT solution (5 mg/mL) (Sigma-Aldrich) for 3 h and colored formazan product was then solubilized in a sodium dodecyl sulfate buffer (10% SDS in 0.01 N HCl) overnight. The absorbance of samples was measured at 570 nm using Infinite M200 Pro microplate reader (Tecan, Männedorf, Switzerland). Cell viability was expressed as a percentage relative to the untreated control cells. The final concentration of DMSO in cell culture did not exceed 0.1% (*v*/*v*) and it did not affect cell viability in comparison to the untreated control.

### 4.8. Cell Proliferation Assay

The evaluation of proliferation of gastric cancer cells was performed using the commercially available BrdU Cell Proliferation ELISA Kit (Roche Diagnostics, Mannheim, Germany). This assay detects BrdU incorporation into newly synthesized DNA of actively proliferating cells in place of thymidine. The ACC-201 and NCI-N87 cells were plated in 96-well plates at an optimal density and treated with alkaloids for 72 h. Next, following the manufacturer’s instructions, the anti-BrdU antibody bound to BrdU was detected by an anti-mouse horseradish peroxidase (HRP)-linked secondary antibody and tetramethylbenzidine (a HRP substrate). The measurement of absorbance values was performed at 450 nm using Infinite M200 Pro microplate reader (Tecan).

### 4.9. Cell Cycle Analysis

Flow cytometry was used to analyze the cell cycle distribution. Optimized amounts of ACC-201 (2 × 10^4^/mL) and NCI-N87 (1 × 10^5^/mL) cells were seeded in 6-well plates (Nunc). After 24 h, cells were cultured in the absence (control) or presence of **11**. After substance exposure time, detached cells were fixed in ice-cold 80% ethanol at −20 °C for 24 h. After that, cells were washed in PBS prior to staining with propidium iodide utilizing PI/RNase Staining Buffer (BD Biosciences, Heidelberg, Germany). The DNA content was determined by a FACS Calibur^TM^ flow cytometer (BD Biosciences) (for more details see [78]).

### 4.10. Active Caspase-3/7 Apoptosis Assay

The quantitation of apoptotic cells was determined by flow cytometry. Optimized amounts of ACC-201 (2 × 10^4^/mL) and NCI-N87 (1 × 10^5^/mL) cells were cultivated in 6-well plates (Nunc) for 24h. After **11** treatments, cells were rinsed with PBS and then the apoptosis assay was performed according to the manufacturer’s instruction of the Caspase 3/7 Staining Kit (Far Red) (Abcam, Fremont, CA, USA).

### 4.11. The Pharmacological Interaction between Coptisine and CDDP with Isobolographic Analysis

An experimental, mathematical-statistical model, such as the isobolographic analysis was used to characterize pharmacodynamic interaction between **11**, the most active alkaloid, and CDDP. Log-probit analysis, according to Litchfield and Wilcoxon [41], was used to determine the percentage of inhibition of cell viability per dose of CDDP and **11** when administered singly in the ACC-201 and NCI-N87 cell lines measured in vitro by the MTT assay. Subsequently, from the log-probit dose–response lines, median inhibitory concentrations (IC_50_ values) of CDDP and **11** were calculated as described earlier [48]. Test for parallelism between two dose–response curves (CDDP and **11**) was performed according to the log-probit method, as described in detail in our previous studies [79,80,81]. Interactions between CDDP and **11** in ACC-201 and NCI-N87 cancer cell lines were isobolographically analyzed as described elsewhere [81,82,83,84]. The median additive inhibitory concentrations (IC_50add_) for the mixture of CDDP with **11**, which theoretically should inhibit 50% of cell viability, were calculated as demonstrated by Tallarida [83,84]. The assessment of the experimentally-derived IC_50mix_ at the fixed-ratio of 1:1 was based on the concentration of the mixture of CDDP and **11** that inhibited 50% of cell viability in both, ACC-201 and NCI-N87 cancer cell lines measured in vitro by the MTT assay. To calculate the concentrations of particular drugs (CDDP and **11**) in the mixture, the IC_50mix_ values were multiplied by the proportions of CDDP and **11**. Details concerning the isobolographic analysis have been published elsewhere [48,81,82,83].

### 4.12. Zebrafish Experiments

To assess the effect of **11** on developing organisms, the zebrafish embryo acute toxicity study was carried out based on the OECD, as mentioned (Test NO. 236) [42].

For all zebrafish experiments, compliance with the National Institute of Health Guidelines for the Care and Use of Laboratory Animals, the European Community Council Directive of November 2010 for Care and Use of Laboratory Animals (Directive 2010/63/EU) guideline was adhered to. Even though the ethical permission is not required for experiments with embryos and larval zebrafish up to 120 hpf, all efforts were made to minimize the number of animals used as well as their suffering. Immediately after the experiments, zebrafish were euthanized with the aid of 15 µM tricaine.

Toxicity studies were performed on zebrafish embryos of the AB strain, purchased from the Experimental Medicine Centre (Medical University of Lublin, Poland). Zebrafish embryos and larvae were housed under standard conditions (28.5 °C, 14-h light/10-h dark cycle) in an incubator [85]. For the purpose of our experiments, larvae up to 96 hpf were used.

One hour after fertilization, zebrafish embryos were selected—only fertilized and completely transparent embryos were transferred to 48 well plates. In each well, at least 3 embryos were kept in the 400 µL of medium without, or supplemented with, different doses of **11** (12.5, 25, 62.5, 125, 187.5 or 250 µg/mL) (39.02, 78.04, 195.12, 390.24, 585.37 or 780.49 µM). Zebrafish embryos were incubated in the **11** solutions from 1 hpf until 96 hpf. The absorbance of **11** by zebrafish were evaluated in 96 hpf-old larvae (n = 100 larvae/sample, 4–6 samples/concentration) by HPLC-MS using the same chromatographic method as described above in Section 4.4. For this purpose, the larvae were homogenized using a laboratory homogenizer with the addition of acetonitrile: water (50:50 *v*/*v*) mixture. The obtained homogenate was later centrifuged at 20,000 rpm for 20 min and the supernatant was taken, filtered through a nylon syringe filter (nominal pore size of 0.1 µm diameter) and subjected to HPLC-MS analysis in the positive ionization mode.

To evaluate the toxicity of different doses of **11**, the following phenotypic traits were scored: (1) hatchability at 72 and 96 hpf, (2) morphological abnormalities at 96 hpf, and (3) escape response at 96 hpf. For morphological phenotyping, the following parameters were scored: pericardial oedema, jaw development, yolk sac necrosis, swim bladder development, body axis shape/curvature, and hemorrhage. For representative images, 96 hours-old larvae were mounted and photographed [85]. The escape response was evaluated, according to our previously described method [86], to determine whether **11** may affect muscle performance and function [87].

### 4.13. Statistical Analysis

Statistical analysis was performed based on one-way analysis of variance (one-way ANOVA) followed by Tukey’*spost-hoc* test using GraphPad Prism 6 or 9 Statistic Software. Data were expressed as the mean ± standard deviation (mean ± SD) (* *p*< 0.05, ** *p*< 0.01, *** *p*< 0.001, **** *p*< 0.0001). The IC_50_ and IC_50exp_ values for **11** and CDDP administered alone or in combination at the fixed-ratio of 1:1 were determined by means of log-probit linear regression analysis, according to Litchfield and Wilcoxon [41]. The unpaired Student’s *t*-test, according to Tallarida [88], was used for statistical comparison of IC_50exp_ values for the mixture of **11** with CDDP with their corresponding IC_50add_ values. In case of all zebrafish experiments, the measurements/scores were replicated three times and the data were pooled together. Here, Chi-squared test or Fisher’s exact test were used for statistical purposes.

## Figures and Tables

**Table 1:** Tentatively identified major alkaloids in the methanolic extract from *Coptis chinensis* by HPLC-ESI-QTOF-MS/MS analysis (Rt—retention time, DBE—double bond equivalent, error—error of measurement).

No	Ion (+/−)	Rt (min)	Molecular Formula	*m/z* Calculated	*m/z* Experimental	Error (ppm)	DBE	MS/MS Fragments	Proposed Compound	References
**1**	[M + H]^+^	6.6	C_20_H_24_NO_4_^+^	342.1700	342.1733	−9.72	10	297, 265, 237	Magnoflorine	This study, [25]
**2**	[M + H]^+^	8.9	C_19_H_23_NO_3_	314.1751	314.1777	−8.4	9	269, 237	4′-*O*-Methyl-N-methylcoclaurine	This study, [26]
**3**	[M + H]^+^	10.1	C_21_H_26_NO_4_	356.1856	356.1888	−8.9	10	206, 175	Menisperine	This study, [25]
**4**	[M + H]^+^	10.5	C_21_H_25_NO_5_	372.1805	372.1833	−7.41	10	357, 222, 162	Stecepharine	This study, [25]
**5**	[M + H]^+^	10.7	C_19_H_17_NO_4_	324.1230	324.1245	−4.54	12	309, 294, 266	Demethyleneberberine/isomer	This study, [25]
**6**	[M + H]^+^	10.85	C_19_H_16_NO_4_^+^	322.1074	322.1100	−8.15	13	250, 192	Berberrubine/Thalifendine	[27]
**7**	[M + H]^+^	11.4	C_20_H_17_NO_5_^+^	352.1153	352.1190	−2.99	13	336, 322, 308, 294	13-methyljatrorrhizine/13-methylcolumbamine	This study, [25]
**8**	[M + H]^+^	11.7	C_20_H_20_NO_4_	338.1413	338.1387	−7.76	12	322, 308, 294, 280	Columbamine	This study, [25]
**9**	[M + H]^+^	11.95	C_20_H_18_NO_4_	336.1230	336.1239	−2.58	13	320, 292, 280, 262	Epiberberine	This study, [25,28]
**10**	[M + H]^+^	12.2	C_20_H_19_NO_4_	338.1387	338.1415	−8.35	12	322, 307, 294, 280	Jatrorrhizine	This study, [25]
**11**	[M + H]^+^	12.6	C_19_H_13_NO_4_	320.0917	320.0942	−7.73	14	320, 292, 277, 262	Coptisine	This study, [25]
**12**	[M + H]^+^	13.1	C_21_H_21_NO_4_	352.1543	352.1575	−9.01	12	337, 322, 308, 291	Palmatine	This study, [25]
**13**	[M + H]^+^	13.5	C_20_H_17_NO_4_	336.1230	336.1256	−7.66	13	321, 306, 292, 275	Berberine	This study, [25]
**14**	[M + H]^+^	17.0	C_21_H_19_NO_4_	350.1387	350.1415	−8.06	13	335, 320, 306, 292	13-Methylberberine	This study, [25]
**15**	[M + H]^+^	10.6	C_20_H_15_NO_4_	334.1074	334.1075	−0.35	14	319, 304, 290, 276	Methylcoptisine	This study, [29]
**16**	[M + H]^+^	8.6	C_20_H_23_NO_4_	342.1700	342.1668	9.33	10	297, 285, 265, 188	Phellodendrine	This study, [30]
**17**	[M + H]^+^	9.8	C_20_H_21_NO_4_	340.1543	340.1576	−9.63	11	325, 308, 192	Tetrahydroberberine (canadine)	This study, [30]
**18**	[M + H]^+^	16.5	C_20_H_15_NO_4_	334.1074	334.1077	−0.95	14	321, 304, 292, 278	Worenine	This study, [19]

**Table 2:** IC_50_ values (measured using MTT assay) of different isoquinoline alkaloids isolated from *Coptis chinensis* against human gastric cancer cells.

Compound	IC_50_ Value ± S.E.M.	
	ACC-201 Cells	NCI-N87 Cells
Berberine (**13**)	0.999 µg/mL (2.97 µM) ± 0.013	2.023 µg/mL (6.01 µM) ± 0.016
Coptisine (**11**)	1.260 µg/mL (3.93 µM) ± 0.250	2.110 µg/mL (6.58 µM) ± 0.750
Jatrorrhizine (**10**)	12.15 µg/mL (35.90 µM) ± 0.016	17.85 µg/mL (52.75 µM) ± 0.018
Palmatine (**12**)	4.909 µg/mL (13.93 µM) ± 0.017	20.08 µg/mL (56.98 µM) ± 0.017

**Table 3:** Type I isobolographic analysis of interactions for parallel concentration–response relationship lines between coptisine (**11**) and CDDP at the fixed drug concentration ratio of 1:1 in ACC-201 cell line. Results are median inhibitory concentrations (IC_50_ values in μg/mL ± S.E.M.) for two-drug mixtures, determined either experimentally (IC_50exp_) or theoretically calculated (IC_50add_) from the equations of additivity, blocking proliferation in 50% of tested ACC-201 cells measured in vitro by the MTT assay. ** *p* < 0.01 vs. the respective IC_50add_ value.

IC_50exp_ (μg/mL)	n_exp_	IC_50add_ (μg/mL)	n_add_	Interaction
0.38 ± 0.09	96	1.13 ± 0.23 **	236	Synergy

**Table 4:** Type I isobolographic analysis of interactions for non-parallel concentration–response relationship lines between coptisine (**11**) and CDDP at the fixed drug concentration ratio of 1:1 in NCI-N87 cell line. Results are median inhibitory concentrations (IC_50_ values in μg/mL ± S.E.M.) for two-drug mixtures, determined either experimentally (IC_50exp_) or theoretically calculated (IC_50add_) from the equations of additivity, blocking proliferation in 50% of tested NCI-N87 cells measured in vitro by the MTT assay. L-IC_50_—lower additive IC_50_ value, U-IC_50_—upper additive IC_50_ value.

IC_50exp_ (μg/mL)	n_exp_	L-IC_50add_ (μg/mL)	n_add_	U-IC_50add_ (μg/mL)	Interaction
2.476 ± 0.550	96	0.824 ± 0.367	236	1.291 ± 0.944	Additivity

## Data Availability

The supplementary file shows all data produced in the course of this study.

## Supplementary Materials

The supporting information can be downloaded at: https://www.mdpi.com/article/10.3390/ijms231810330/s1.
