# Supplementary material for: Isoquinoline Alkaloids from Coptis chinensis Franch: Focus on Coptisine as a Potential Therapeutic Candidate against Gastric Cancer Cells
Source: Int J Mol Sci. 2022 Sep 7;23(18):10330. doi: 10.3390/ijms231810330 (PMC9499618; doi:10.3390/ijms231810330)
Supplement: Supplementary file 1 [file ijms-23-10330-s001.zip › ijms-1864517-supplementary.pdf]

Supplementary file

Isoquinoline alkaloids from *Coptis chinensis* Franch: focus on coptisine as a potential therapeutic candidate against gastric cancer cells

Sylwia Nakonieczna [1,∆], Aneta Grabarska [2,∆,*], Kinga Gawel [3], Paula Wróblewska – Łuczka [4], Arkadiusz Czerwonka [2], Andrzej Stepulak [2] and Wirginia Kukula-Koch [1,*]

Figure S1. The UV (at 290 nm) chromatogram of the methanolic extract from the rhizomes of *Coptischinensis*

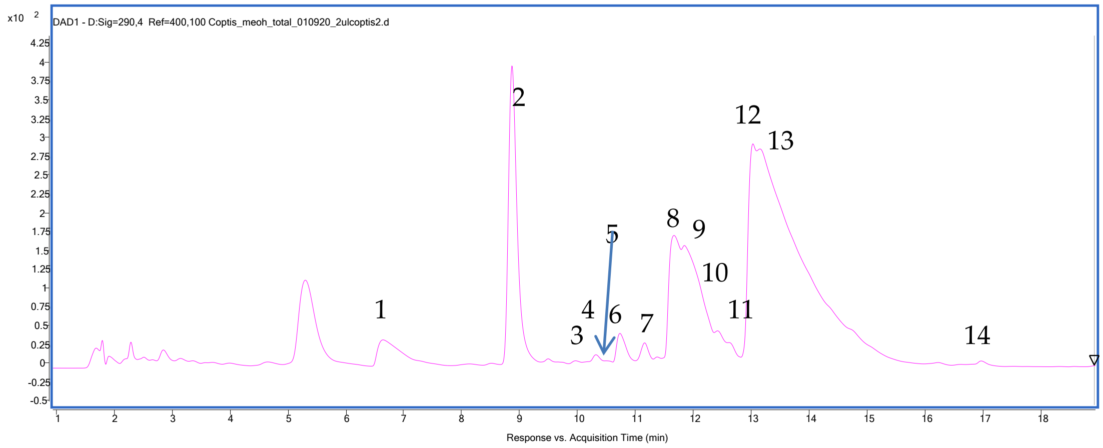

Table S1. The list of MS/MS spectra of the tentatively identified compounds

Name of the compound	MS/MS spectrum
**Magnoflorine**	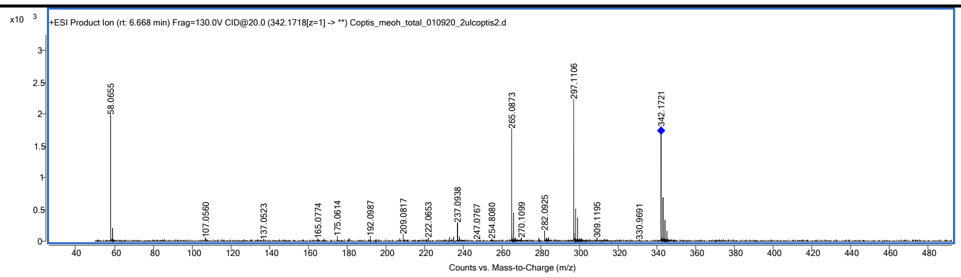

**4′-O-Methyl-N-methylcoclaurine**	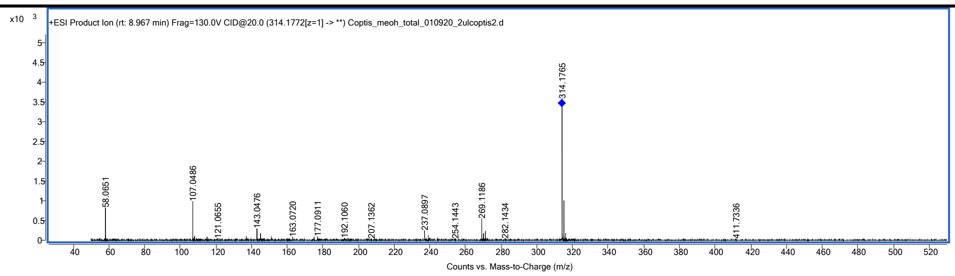
**Menisperine**	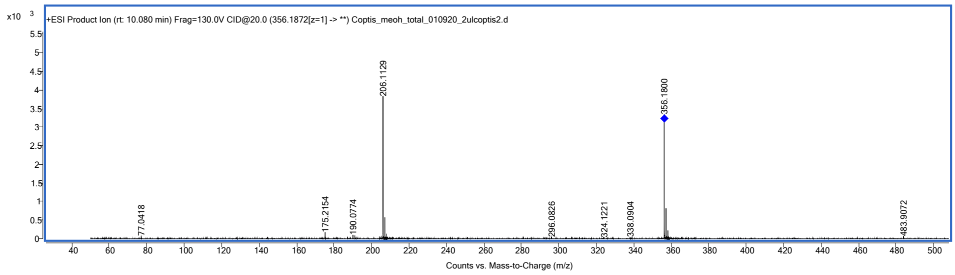
**Stecepharine**	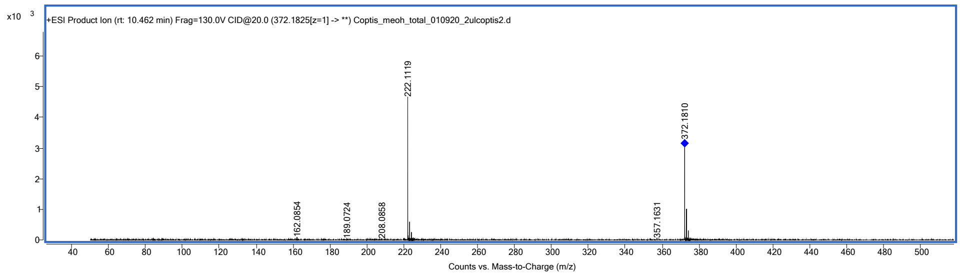
**Demethylene berberine/isomer**	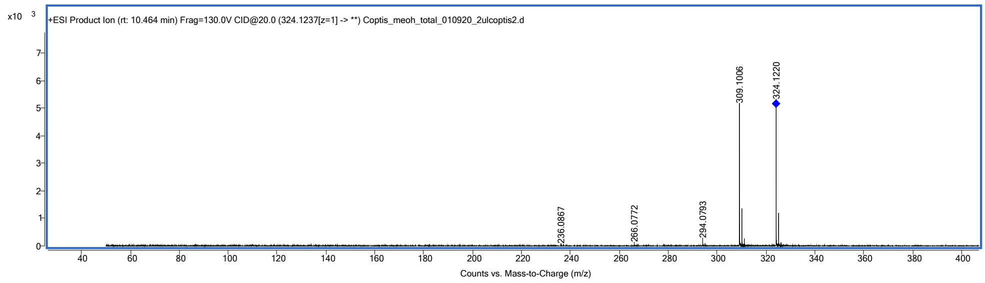
**Berberrubine /Thalifendine**	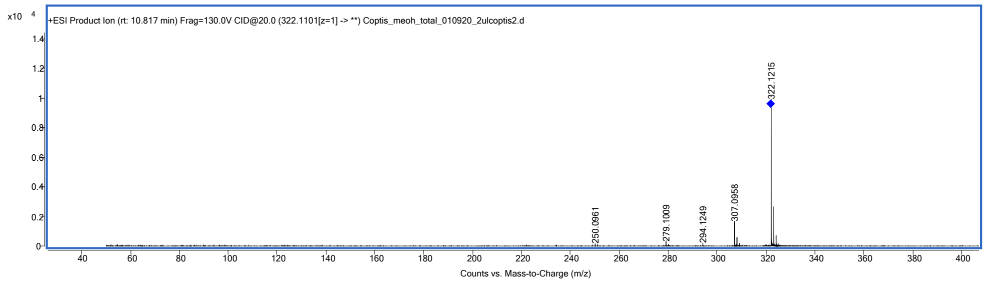

**13-methyljatrorrhizine/13-methylcolumbamine**	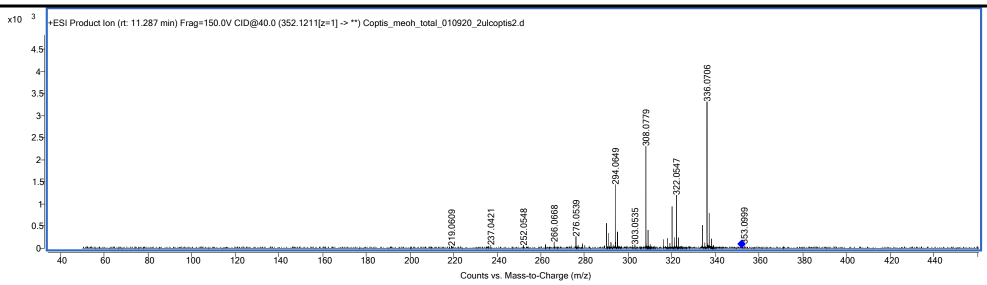
**Columbamine**	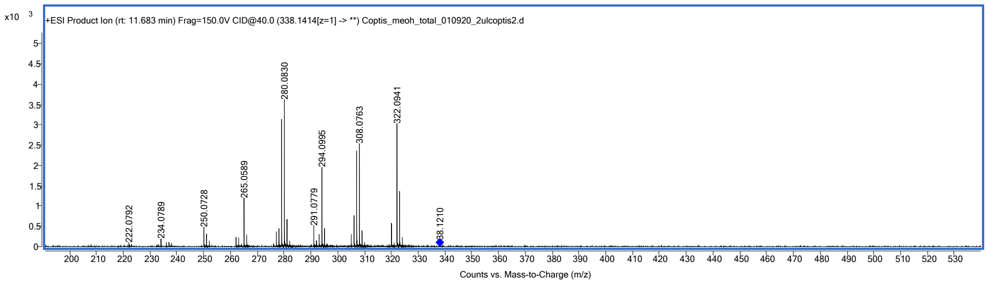
**Epiberberine**	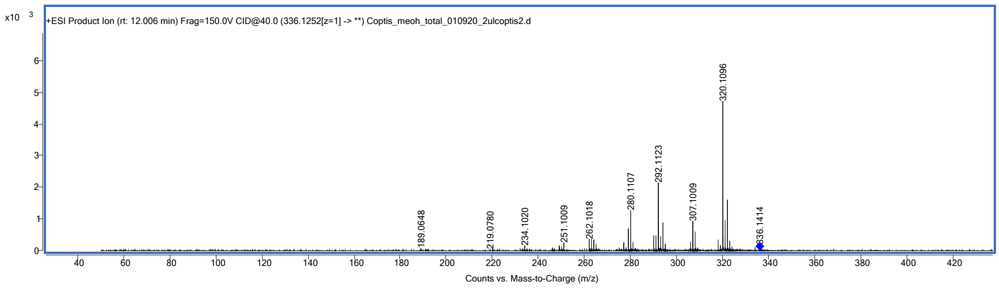
**Jatrorrhizine**	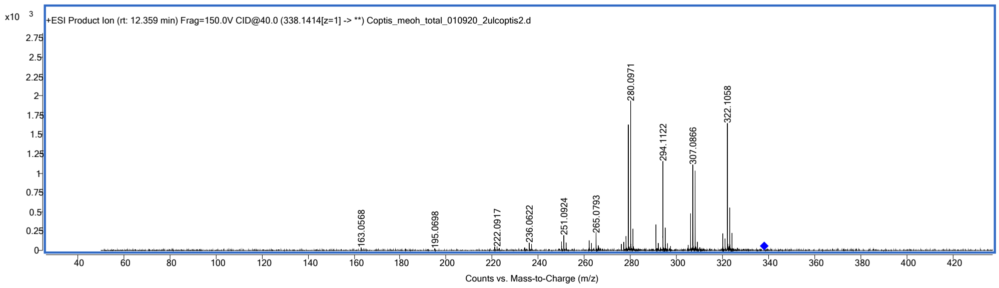
**Coptisine**	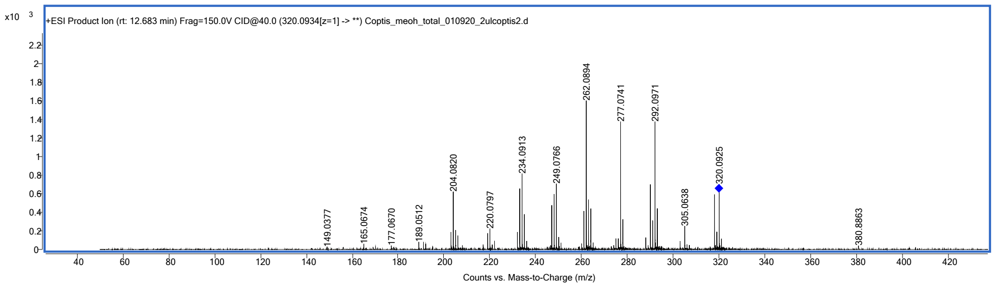

Palmatine

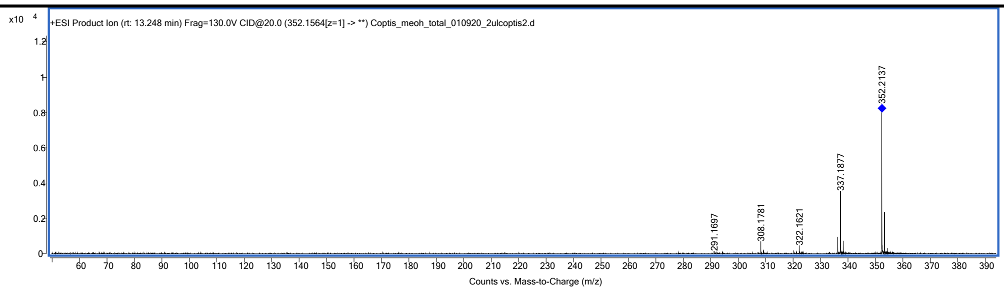

Berberine

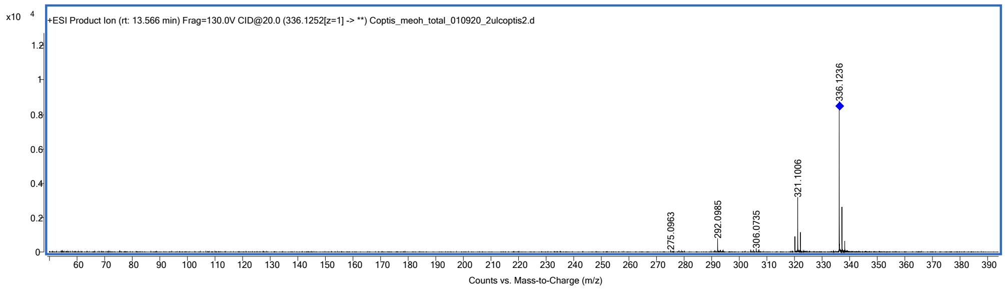

13-Methylberberine

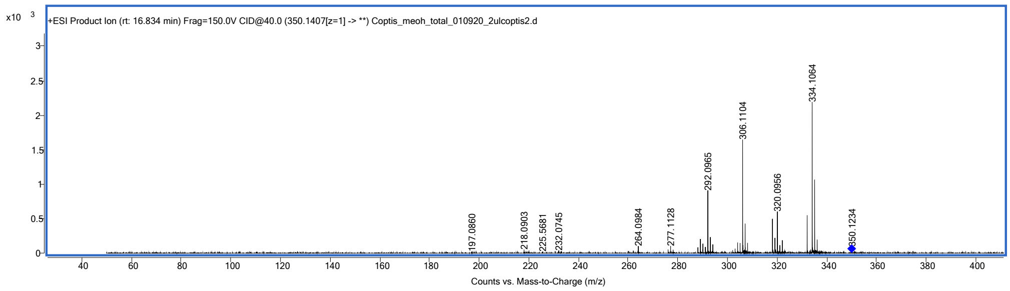

Dihydrosanguinarine

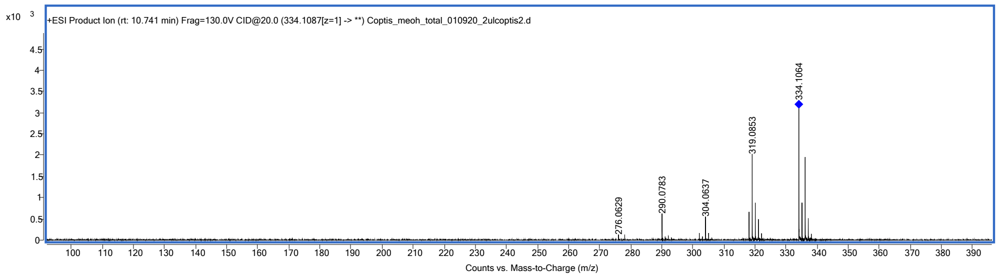

Phellodendrin

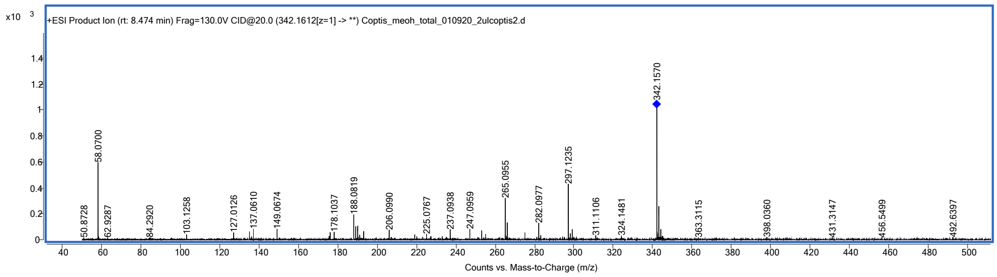

**Tetrahydroberberine**	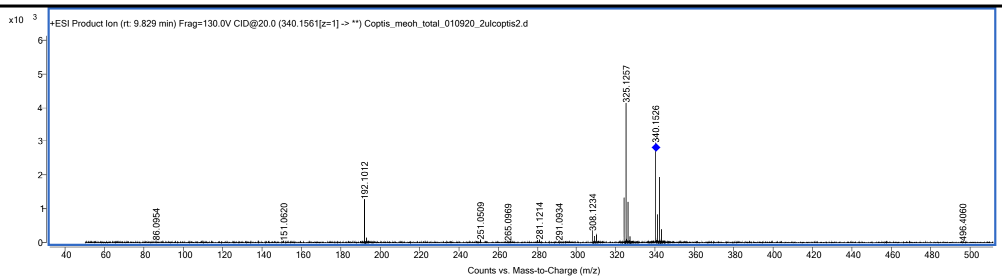
**Worenine**	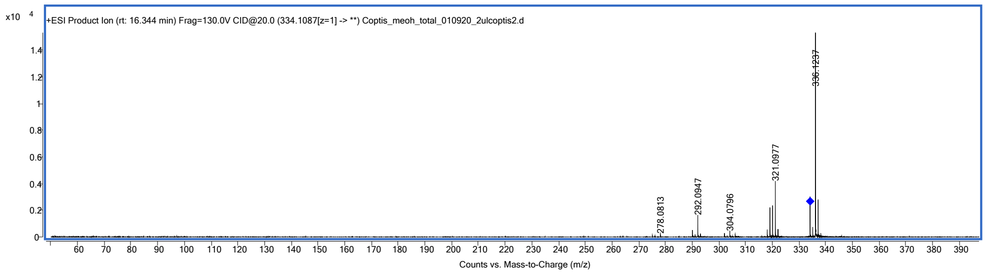
